# Accelerated probabilistic inference of RNA structure evolution

**DOI:** 10.1186/1471-2105-6-73

**Published:** 2005-03-24

**Authors:** Ian Holmes

**Affiliations:** 1 Department of Bioengineering, University of California, Berkeley CA 94720-1762, USA

## Abstract

**Background:**

Pairwise stochastic context-free grammars (Pair SCFGs) are powerful tools for evolutionary analysis of RNA, including simultaneous RNA sequence alignment and secondary structure prediction, but the associated algorithms are intensive in both CPU and memory usage. The same problem is faced by other RNA alignment-and-folding algorithms based on Sankoff's 1985 algorithm. It is therefore desirable to constrain such algorithms, by pre-processing the sequences and using this first pass to limit the range of structures and/or alignments that can be considered.

**Results:**

We demonstrate how flexible classes of constraint can be imposed, greatly reducing the computational costs while maintaining a high quality of structural homology prediction. Any score-attributed context-free grammar (e.g. energy-based scoring schemes, or conditionally normalized Pair SCFGs) is amenable to this treatment. It is now possible to combine independent structural and alignment constraints of unprecedented general flexibility in Pair SCFG alignment algorithms. We outline several applications to the bioinformatics of RNA sequence and structure, including Waterman-Eggert N-best alignments and progressive multiple alignment. We evaluate the performance of the algorithm on test examples from the RFAM database.

**Conclusion:**

A program, Stemloc, that implements these algorithms for efficient RNA sequence alignment and structure prediction is available under the GNU General Public License.

## Background

As our acquaintance with RNA's diverse functional repertoire develops [1-5], so does demand for faster and more accurate tools for RNA sequence analysis. In particular, comparative genomics approaches hold great promise for RNA, due to the well-behaved basepairing correlations in an RNA gene family with conserved secondary structure (at least, well-behaved compared to protein structures). Whereas the structural signal encoded in a single RNA gene is rather weak and may be barely (if at all) distinguishable from the secondary structure of a random sequence [6], the covariation signal increases with every additional sequence considered.

Many programs for comparative analysis of RNA require the sequences to be prealigned [7-9]. This can be a source of error, since misaligned bases can add noise that swamps the covariation signal. The most recent of these methods allows for some uncertainty in the alignment [7]. More generally, one can view the alignment and structure prediction as a combined problem, to be solved simultaneously. This is the approach taken in this paper, and by earlier programs such as FOLDALIGN [10], DYNALIGN [11], CARNAC [12], QRNA [9] and our dart library, introduced in a previous paper [13] and extended here. In this framework, fixing of the alignment can be viewed as a partial constraint on the simultaneous alignment/folding problem.

A powerful, general dynamic programming algorithm for simultaneously aligning and predicting the structure of multiple RNA sequences was developed by David Sankoff [14]. The energy-based folding of Zuker *et al *[15] and recent approaches based on Stochastic Context-Free Grammars (SCFGs) [9,13,16-20] are both closely related to Sankoff's algorithm. The method takes time *O*(*L*^3*N*^) and memory *O*(*L*^2*N*^) for *N *sequences of length *L*. This is prohibitively expensive at the time of writing, except for fairly short sequences, which has motivated the development of various constrained versions of these algorithms [9-11,13,21].

The purpose of this paper is to report our progress on general pairwise constrained versions of Sankoff's algorithm (or, more precisely, constrained versions of some related dynamic programming algorithms for SCFGs). The overall aim is the simultaneous alignment and structure prediction of two RNA sequences, *X *and *Y*, subject to an SCFG-based scoring scheme and user-supplied constraints. Additionally, we wish to be able to parameterize the model automatically from training data. Without constraints, the above tasks are addressed by the resource-intensive CYK and Inside-Outside algorithms; here, we present constrained versions of these algorithms that work in reduced space and time (the exact complexity depends nontrivially on the constraints).

Our system of constraints is quite general. Previous constrained versions of Sankoff-like algorithms, such as the programs DYNALIGN [11] and FOLDALIGN [10], have been restricted to "banding" the algorithm e.g. by constraining the maximum insertion/deletion distance between the two sequences or the maximum separation between paired bases. Alternately, constraints on the accessible structures [13] or alignments [9] have been described.

The algorithms described here can reproduce nearly all such banding constraints and, further, can take advantage of more flexible sequence-tailored constraints. Specifically, the *fold envelopes *determine the *subsequences *of *X *and *Y *that can be considered by the algorithm, while the *alignment envelope *determines the permissible *cutpoints *in the pairwise alignment of *X *and *Y*. The fold envelopes can be used to prune the search over secondary structures (e.g. by including/excluding specific hydrogen-bonded base-pairings), while the alignment envelopes can be used to prune the search over alignments (e.g. by including/excluding specific residue-level homologies). The fold envelopes can be precalculated for each sequence individually (e.g. by an energy-based folding or a single-sequence SCFG), and the alignment envelope by comparing the two sequences without regard for secondary structure (e.g. using a pairwise Hidden Markov Model); both types of pre-comparison are much more resource-friendly than the unconstrained Sankoff-like algorithms. The design of the constrained algorithms is discussed using concepts from object-oriented programming: the dynamic programming matrix can be viewed as a sparsely populated *container*, whereas the main loop that fills the matrix is a complex *iterator *[22]. The algorithms have been implemented in a freely available program for RNA sequence alignment, stemloc, which also includes algorithms to determine appropriate constraints in an automatic fashion. Results demonstrating the program's efficient resource usage are presented.

The stemloc program also implements various familiar extensions to pairwise alignment, including local alignment [23], Waterman-Eggert *N*-best suboptimal alignments [24] and progressive multiple alignment [25]. Although the envelope framework, rather than these extensions, is the main focus of this paper, implementation of the extensions is straightforward within this framework, and is briefly described.

## Results

To investigate the comparative resource usage of the various different kinds of constraint that can be applied using fold and alignment envelopes, stemloc was tested on 22 pairwise alignments taken from version 6.1 of RFAM [37], spanning 7 different families of functional noncoding RNA. Each chosen test family had a consensus secondary structure published independently in the literature, and no two sequences in the test set had higher than 60% identity.

The EMBL accession numbers and co-ordinates of all sequences are listed in Table 5. The table shows the performance of stemloc using the 1000-best fold envelope and the 100-best alignment envelope. The various RFAM families are S15, the ribosomal S15 leader sequence; the U3 and U5 spliceosomal small nucleolar RNAs; IRE, the iron response element from UTRs of genes involved in vertebrate iron metabolism; glmS, the glucosamine-6-phosphate activated mRNA-cleaving ribozyme; Purine, the prokaryotic purine-binding riboswitch; and 6S, the *E.coli *polymerase-associated transcriptional repressor. The following three test regimes were used, each representing a different combination of fold and alignment envelopes:

### *N*-best alignments, all folds

The alignment envelope containing the *N *best primary sequence alignments, with the unconstrained fold envelopes (stemloc options: '--nalign N --nfold -1'). This is the red curve in Figures 8, 9, 10, 11, 12, 13

### *N*-best folds, all alignments

The unconstrained alignment envelope, with the fold envelopes containing the *N *best single-sequence structure predictions (stemloc options: '--nalign -1 --nfold *N*'). This is the green curve in Figures 8, 9, 10, 11, 12, 13

### *N*-best folds, 100-best alignments

The alignment envelope containing the 100 best primary sequence alignments, with the fold envelopes containing the *N *best single-sequence structure predictions (stemloc options: '--nalign 100 --nfold *N*'). This is the blue curve in Figures 8, 9, 10, 11, 12, 13

In the first two tests, *N *was varied from 1 to 100; in the latter test, *N *was varied from 1 to 10000. The lower ceiling for *N *in the first two tests was imposed by resource limitations. Note that the endpoint of the red curve ("*N*-best alignments, all folds"), which occurs at *N *= 100, coincides with the asymptotic limit of the blue curve ("*N*-best folds, 100-best alignments") at high *N*.

A range of different values for the parameter *N *was used to test the above three strategies. As *N *was increased over the range, the size of the corresponding fold or alignment envelopes was found to be strongly correlated (Figures 6, 7). However, the actual size of the fold/alignment envelopes in each particular test case varies widely (see large error bars in Figures 6, 7), perhaps due to variable factors such as the sequence lengths, compositions and/or identities. Since it is easier to control the envelope construction parameter *N *than to control the envelope sizes directly, the following section will report performance indicators as a direct function of *N*, rather than as a function of the strongly-correlated but widely-varying envelope sizes. We report performance indicators for stemloc as follows. Let *A *and *B *be alignments of a given pair of sequences, each represented as a set of aligned residue-pairs {(*i*, *k*)}. Suppose that *A *is the alignment according to RFAM, and *B *is the alignment predicted by stemloc. Then define the *alignment sensitivity *to be |*A *∩ *B*|/|*A*| and the *alignment specificity *to be |*A *∩ *B*|/|*B*|. Further, let *S *and T be possible secondary structures for a given sequence, each represented as a set of base-pairs {(*i*, *j*)}. Suppose that *S *is the published structure, and T is the structure predicted by stemloc. Then the *basepair sensitivity *is |*S *∩ *T*|/|*S*| and the *basepair specificity *is |*S *∩ *T*|/|*T*|.

These performance indicators are averaged over all 22 pairwise alignments and plotted for the three test regimes in Figure 8 (alignment sensitivity), Figure 9 (alignment specificity), Figure 10 (basepair sensitivity) and Figure 11 (basepair specificity). As can be seen, the *N *-best alignment regime empirically seems to achieve an asymptotic maximum performance around *N *≃ 100 (possibly even around *N *≃ 10), while the *N *-best fold envelope underperforms compared to the unconstrained fold envelope up to around *N *≃ 1000. The tests were performed on a 2.3 GHz Apple PowerPC G5. The resource usages of the test regimes are plotted in Figure 12 (user-mode running time) and Figure 13 (memory usage). The resource usage of the constrained algorithms is substantially reduced when the envelopes are smaller (i.e. at lower *N *). This is especially notable when contrasting the resource usage of the "*N *-best folds, all alignments" test with the more constrained "*N *-best folds, 100-best alignments" test.

Three main conclusions can be drawn from these data. First, allowing the search to consider more than a single alignment greatly improves structure prediction (the red curve). Second, constraining the alignment search while exhaustively scanning fold space (the red curve) outperforms constraining the fold search while exhaustively scanning alignment space (the green curve). Third, the hybrid strategy (the blue curve), which partially constrains both searches, approaches the alignment-constrained, fold-unconstrained strategy (the red curve) in performance, with a significant saving in CPU and memory resources. Memory is the limiting factor in pairwise RNA alignment, and the primary motivation for constraints. For example, without constraints, alignment of two 16S ribosomal subunits using the stemloc grammar would take approximately 500 terabytes. (Using fold envelope constraints with structures fully specified, it can be done in under 5 gigabytes.)

Based on the results of these tests, the default envelope options for stemloc were chosen to be the 100-best alignment envelope and the 1000-best fold envelope. The performance of stemloc with these envelopes on each of the pairwise test alignments is given in Table 5.

## Discussion

The algorithms presented here include constrained versions of Pair-SCFG dynamic programming algorithms that run in significantly reduced space and time. The primary advance over previous work is the simultaenous imposition of fold and alignment constraints, including alignment constraints that are more general than others previously described. Thes constraints lead to significant reductions in requirements for processor and memory usage, which will increase the length of RNA sequences that can be analyzed on mainstream computer hardware.

These algorithms have been used to implement stemloc, a fast, efficient software tool for multiple RNA sequence alignment implementing numerous extra features such as local alignment, Waterman-Eggert *N *-best suboptimal alignment and progressive multiple alignment. The source code for the program is freely available from .

The results given here should be regarded as preliminary. For example, we have only tested the pairwise alignment functionality; full evaluation/optimisation of the multiple alignment algorithm remains. Rather than using the CYK algorithm, one could use the Inside-Outside algorithm with a decision-theoretic dynamic programming step to maximize expected performance [38,39]. As noted in the Parameterization section, it might also be possible to improve on the training procedure. We are also considering ways of elaborating the grammar to include basepair stacking terms. These and other improvements we hope to address in future work.

## Conclusion

RNA sequence analysis has generated considerable interest over recent years, as many new roles for RNA in the cell have come to light. RNA genes and regulatory elements are components of many molecular systems and comparative genomics is a powerful way to probe this function, perhaps even more so for RNA than for protein (due to the "well-behaved" statistical correlations found in RNAs with conserved secondary structure). Furthermore, statistical modeling of RNA evolution continues to play a fundamental role in the phylogenetic classification of new forms of life.

These biological motives have driven a demand for RNA sequence analysis tools that are faster, slimmer and more scaleable. It is hoped that the algorithms and approaches described here, together with development and analysis of RNA evolutionary models [36], may expand the applications of RNA informatics.

## Methods

We begin our description of the envelope method with an explanatory note regarding our decision to present these constraints in terms of SCFGs, rather than other scoring schemes such as those based solely on energies [15] or on energy/information-theoretic hybrids [11].

The reason for our choice of SCFGs is simple: stochastic grammars are, in our opinion, the most theoretically well-developed of the scoring schemes used for RNA. They come with well-documented algorithms for sequence alignment, structure prediction, parameterization by supervised learning from various kinds of training data, and calculation of posterior probabilities [20]. Discussion of these algorithms is facilitated by a well-developed and widely-understood probabilistic vocabulary. Stochastic grammars are actively researched outside bioinformatics, principally in natural language processing [26]. Crucially, SCFGs are sufficiently general to express virtually all of the features offered by other scoring schemes [19]. We also acknowledge the appeal of free energy-based scoring schemes, which have the advantage that the parameters can be determined experimentally. Energy-based scores can also be used to find posterior probabilities of base-pairings using a partition function [27]. However, to extend energy-based methods to two or more sequences, one must incorporate substitution scores. These are information-theoretic in nature and so are measured in bits, rather than kilocalories-per-mole [28]. Reconciling these two units of score (in a principled way) is an open problem. However, in an SCFG framework, all scores are information-theoretic and so there is no conflict of units.

Despite these arguments, many people continue to find calories preferable to bits as a unit of score. For such readers, we note that the system of constraints described here is entirely applicable to the general score-attributed grammar. This includes energy-based and heuristic scoring schemes as well as (for example) grammars whose rule "probabilities" actually represent log-odds ratios, or which are conditionally normalized with respect to one sequence.

### Notation

To implement SCFG dynamic programming algorithms efficiently for RNA, it is convenient to define a simplified (but universal) template for grammars, similar in principle to "Chomsky normal form" [29,30]. Our "RNA normal form" preserves the RNA-optimized efficiency of the Pair SCFG form presented in an earlier paper [13] (based on a single-sequence form due to Durbin *et al *[20]) by introducing different types of production rule to minimize bifurcations and collect emissions. The form defined here is slightly different from the above forms, in that it classifies only production rules, and not nonterminals, into different types. Let  be the "ungapped RNA alphabet", i.e. the set of four possible nucleotides in RNA. Let  be the "gapped RNA alphabet", i.e. the ungapped RNA alphabet Ω plus the gap symbol . Finally, let Ψ = Ω' × Ω' be the "gapped-pair RNA alphabet", i.e. a Cartesian product of two gapped RNA alphabets. We write Ψ-symbols by vertically stacking pairs of Ω'-symbols, like this  or this .

A pairwise stochastic context-free grammar  in RNA normal form consists of a nonterminal symbol alphabet Φ, a terminal symbol alphabet that is the gapped-pair RNA alphabet Ψ, and (for each nonterminal *L*) a probability distribution over a set of transformation rules (or *production rules*), 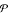
(*L *→ **R**), where **R **= *R*_1 _... *R*_*K *_is a sequence of nonterminal or terminal symbols, taking one of several stereotypical forms (see below). The nonterminal *L *is referred to as the left-hand side (LHS) of the production rule and the symbol sequence **R **as the right hand side (RHS).

Let *S*, *U*, *V*, *W *∈ Φ denote nonterminal symbols. The allowable forms for production rules include **terminations**, **transitions**, **bifurcations **and **emissions**. These are defined as follows

**Terminations**: rules of the form *L *→ *ε*.

**Transitions**: rules of the form *L *→ *V*. The directed graph formed by transition rules on nonterminals must be acyclic, and the list of nonterminals Φ must be topologically reverse-sorted with respect to this graph; i.e. if 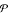
(*U *→ *V*) > 0, then *V *appears before *U *in Φ.

**Bifurcations**: rules of the form *L *→ *VW*. There must be no transition-termination path from *V *or *W *to *ε*, i.e. neither *V *or *W *can have completely empty inside sequence-pairs (see next section for a formal definition of the "inside sequence-pair").

**Emissions**: rules of the form  where *A'*, *B'*, *C'*, *D' *∈ Ω' are gapped RNA symbols, at least one of which is a non-gap symbol. For convenience, we also define *A*, *B*, *C*, *D *∈ Ω* to be the corresponding ungapped RNA sequences, as follows: *A *is the empty string if and only if *A' *is the gap character; otherwise, *A *= *A'*. Similar definitions apply for *B*, *C *and *D*.

The particular RNA normal form described in this section is chosen for ease of presentation. The implementation in the dart library uses the slightly more restrictive form for Pair SCFGs defined in an earlier paper [13].

For presentational purposes, we will generally omit all-gap columns from the pairwise alignment and the grammar. For example, an emission rule having the form  would be written as  instead. All-gap columns are not very interesting to a sequence analyst, and only arise in our formalism because all emission rules have the same form.

Table 1 is an example of an RNA normal form grammar with two nonterminals, Stem and Loop. The grammar generates simple alignments of stems and loops, using two nonterminals (Stem and Loop); the starting nonterminal is Stem. The rule probabilities are functions of five scalar probability parameters (stemExtend, stemGap, bifurcate, loopExtend and loopGap) and four arrays of probability parameters (baseIndel[4], baseSubstitution[16], basepairIndel[16] and basepairSubstitution [256]). Here we introduce the notation X [*N*] for an array of *N *probability parameters normalized so that . It is also convenient to introduce some notation for ungapped sequences at this stage. Let *X*, *Y *∈ Ω* denote ungapped RNA sequences, including (possibly) the empty string *ε*. Let *X*_*i *_be the *i*'th symbol of *X *(counting from 1, so e.g. *X*_3 _is the third symbol), let *X*_*ij *_denote the subsequence from *X*_*i*+1 _to *X*_*j *_inclusive, or the empty string if *i *= *j *(so e.g. *X*_0,3 _contains the first three symbols of *X*, while *X*_3,3 _= *ε*) and let |*X*| denote the length of *X*.

### The parse tree and the sequence likelihood

**Figure 1:**
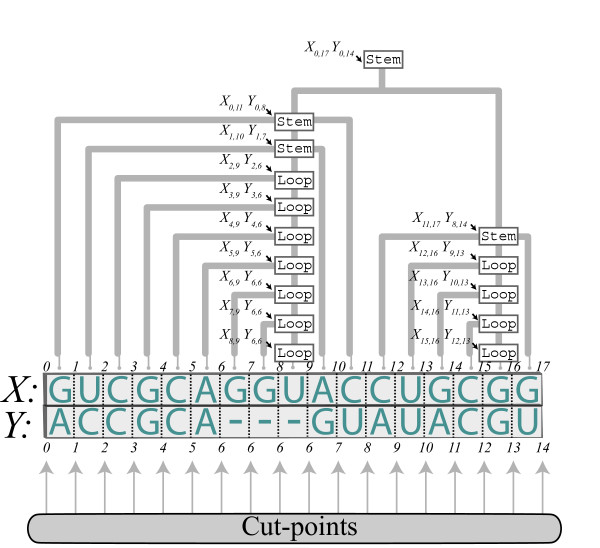
A parse tree for the grammar of Table 1. Each internal node is labeled with a nonterminal (Stem or Loop); additionally, the subsequences (*X*_*ij*_, *Y*_*kl*_) generated by each internal node are shown. The parse tree determines both the structure and alignment of the two sequences. The cut-points of the alignment are the sequence co-ordinates at which the alignment can be split, i.e. {(0, 0), (1, 1), (2, 2) ... (15, 12), (16, 13), (17, 14)}.

The grammar  is a probabilistic model for deriving sequences *X*, *Y *from a single nonterminal. This derivation proceeds as follows: start with an initial sequence containing one starting nonterminal, *S*, then repeatedly apply probabilistically-sampled transformations to the nonterminals in the sequence. Eventually the sequence will contain only terminals from Ψ. This process generates a *parse tree*, rooted at node *S*, in which internal nodes are labeled with nonterminals and leaf nodes with terminals, with children of each node ordered left-to-right (Figure 1). Sequence *X *can be obtained by reading off ungapped RNA symbols from the top row of the output, and sequence *Y *by reading off the bottom row. Note that the subtree rooted at any internal *W*-labeled node describes a sub-process that generates some pair of subsequences (*X*_*ij*_, *Y*_*kl*_) starting from nonterminal *W*. We will refer to this subsequence-pair (*X*_*ij*_, *Y*_*kl*_) as the *inside sequence-pair *of *W*.

The *parse tree likelihood *is the product of all the rule probabilities corresponding to the internal nodes. Summing the likelihoods of all parse trees rooted in state *S *and generating sequences *X*, *Y*, one obtains *P*(*X*, *Y *| *S*,), the *sequence likelihood*. This sum can be performed efficiently by the *Inside algorithm*, as will be described below.

### Dynamic programming algorithms for Pair SCFGs

The following section describes the constrained and unconstrained dynamic programming (DP) algorithms used for Pair SCFGs.

#### The Inside algorithm

The Inside algorithm [26] computes *P*(*X*, *Y *| *S*,) by recursive decomposition via intermediate probabilities (*i*, *j*, *k*, *l*) ≡ *P*(*X*_*ij*_, *Y*_*kl*_|*U*,). In RNA normal form, the time-limiting step in the Inside algorithm involves summing contributions to (*i*, *j*, *k*, *l*) from bifurcation rules of the form *U *→ *VW*, such that the bifurcation splits the two sequences (*X*, *Y*) between bases (*X*_*m*_, *Y*_*n*_) and (*X*_*m*+1_, *Y*_*n*+1_)



An asymptotically faster step involves summing contributions from matching emission rules of the form  (transition rules are represented as the special case *A' *= *B' *= *C' *= *D' *= )



(Recall that *A *is the ungapped version of *A'*. Thus |*A*| = 0 ⇔ *A *= *ε *⇔ *A' *=  and similarly for *B*, *C *and *D*.)

The intermediate probabilities of the Inside algorithm can then be expressed as

(*i*, *j*, *k*, *l*) = *Q*_1 _+ *Q*_2_

Termination of the recursion is provided by matching end rules, *U *→ *ε*, if and only if the subsequences (*X*_*ij*_, *Y*_*kl*_) are empty

(*i*, *i*, *k*, *k*) = 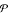
(*U *→ *ε*)

The sequence likelihood is obtained as

*P*(*X*, *Y *| *S*,) ≡ (0,|*X*|,0,|*Y*|)

In pseudocode, the Inside algorithm is

• Inputs: *X*, *Y*, *S*, 

• For *i *= |*X*| to 0 (descending)

    • For *j *= *i *to |*X*|

       • For *k *= |*Y*| to 0 (descending)

          • For *l *= *k *to |*Y*|

       • For each nonterminal *U *∈ *ε*

          • Set *Q*_1 _← 0; calculate *Q*_2_

          • For *m *= *i *to *j*

             • For *n *= *k *to *l*

                • Calculate *Q*_*B*_(*m*, *n*) and add to *Q*_1_

          • Calculate (*i*, *j*, *k*, *l*) and store

• Return (0, |*X*|, 0, |*Y*|)

The time-limiting step of the Inside algorithm (computing the *Q*_*B*_) involves six indices (*i*, *j*, *k*, *l*, *m*, *n*) and the time complexity of the full recursion is *O*(|*X*|^3^|*Y*|^3^). However, the stored intermediate probabilities involve only four indices (*i*, *j*, *k*, *l*) and so the memory complexity is *O*(|*X*|^2^|*Y*|^2^).

**Figure 2:**
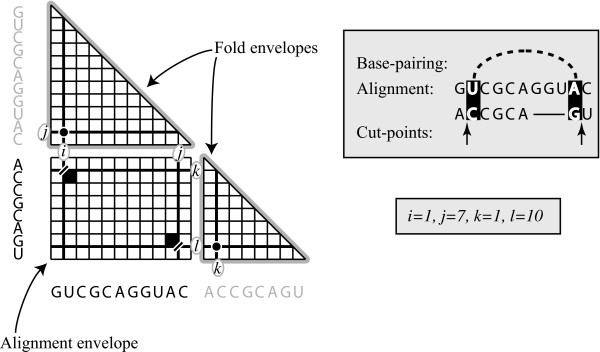
Parsing a pair of sequences (*X, Y*) using the Inside algorithm involves iterating over subsequence-pairs (*X*_*ij*_, *Y*_*kl*_) specified by four indices (*i*, *j*, *k*, *l*). In the constrained Inside algorithm, these indices are only valid if the *fold envelopes *(triangular grids) include the respective subsequences (*i*, *j*) and (*k*, *l*) (shown as black circles) and the *alignment envelope *(rectangular grid) includes both cutpoints (*i*, *k*) and (*j*, *l*) (shown as short diagonal lines). The filled cells in the rectangular grid show the aligned nucleotides. Note that the co-ordinates (*i*, *j*, *k*, *l*) lie on the grid-lines *between *the nucleotides.

**Figure 3:**
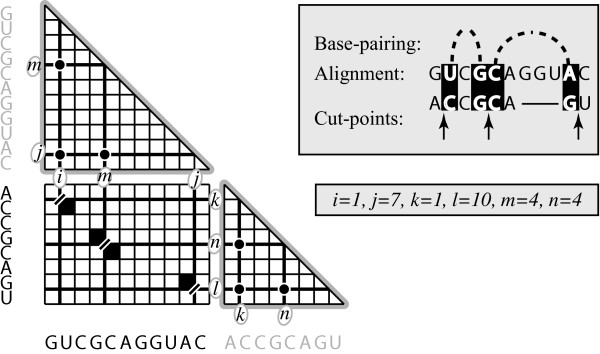
Bifurcation rules allow a subsequence-pair (*X*_*ij*_, *Y*_*kl*_) to be composed from two adjoining subsequence-pairs (*X*_*im*_, *Y*_*kn*_) and (*X*_*nj*_, *Y*_*ni*_). For this to be permitted by the constraints, the *X*-fold envelope (upper triangular grid) must contain subsequences (*i*, *m*), (*m*, *j*) and (*i*, *j*) (black dots), the *Y*-fold envelope (rightmost triangular grid) must contain subsequences (*k*, *n*), (*n*, *l*) and (*k*, *l*) (black dots) and the alignment envelope (rectangular grid) must contain cutpoints (*i*, *k*), (*m*, *n*) and (*j*, *l*) (short diagonal lines). The filled cells in the rectangular grid show the nucleotide homologies highlighted in the alignment. Note that all co-ordinates (*i*, *j*, *k*, *l*, *m*, *n*) lie on the grid-lines between nucleotides.

In RNA normal form, the emission rules (*Q*_2_) account for homologous base-pairings between residues (*X*_*i*_, *X*_*j*_) and (*Y*_*k*_, *Y*_*l*_), or unpaired residues at *X*_*i*_, *X*_*j*_, *Y*_*k *_or *Y*_*l*_. This may also imply that *X*_*i *_and *Y*_*k *_are aligned, or that *X*_*j *_and *Y*_*l *_are (Figure 2). The bifurcation rules (*Q*_*B*_(*m*, *n*)) account for conserved multiloop structures in the RNA, i.e. one homology between substructures *X*_*im *_and *Y*_*kn *_and another between substructures *X*_*mj *_and *Y*_*nl *_(Figure 3).

#### Imposing constraints

The high time and memory cost of the Inside and related algorithms motivate the development of slimmer, faster versions. To begin with, we impose constraints that narrow the search space. For example, we might want to pre-parse the sequences individually (using a single-sequence SCFG, or other *O*(|*X*|^3^) RNA-folding method) and identify likely base-pairs (*X*_*i*_, *X*_*j*_) or (*Y*_*k*_, *Y*_*l*_), and/or likely unpaired nucleotides *X*_*i *_or *Y*_*k*_. Even simpler, we could simply throw out basepairs (*X*_*i*_, *X*_*j*_) between distant residues (i.e. where *j *- *i *exceeds some cutoff) Alternatively, we might want to pre-align the sequences (using a pairwise hidden Markov model, or other *O*(|*X*||*Y*|) alignment method) and identify likely alignment columns (*X*_*i*_, *Y*_*k*_) and/or likely indels *X*_*i *_or *Y*_*k*_. Again, more simply, we could simply exclude columns (*X*_*i*_, *Y*_*k*_) for which |*k *- *i*| is too large.

We can combine these various strategies into a generalized constraint on base-pairs, alignment-columns or both. We stipulate that (*i*, *j*, *k*, *l*) = *P*(*X*_*ij*_, *Y*_*kl*_|*U*, ) = 0 unless the following conditions are satisfied



Here  and  are sets of permissible co-ordinates for structurally discrete subsequences in *X *and *Y*. Fold-related features (basepairs and unpaired residues) can be included or excluded by this set, and so we refer to it as a *fold envelope *[13]. The set  is a set of possible cut-points in the alignment of *X *and *Y*, and is referred to as an *alignment envelope *[31]. Both types of envelope are illustrated in Figure 2. The fold and alignment envelopes satisfy the following set relations



If equality holds in all three cases, then we recover the unconstrained Inside algorithm.

Note that the co-ordinates (*i*, *j*, *k*, *l*) for the cutpoints and subsequences lie *between *residues of *X *and *Y*.

There are (|*X*| + 1)(|*Y*| + 1) cutpoints in the maximal alignment envelope  and (|*X*| + 1)(|*X*| + 2) subsequences in the maximal fold envelope .

**Figure 4:**
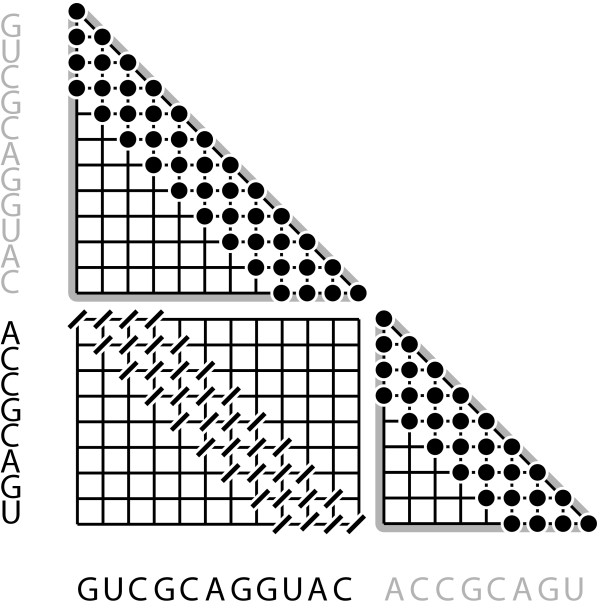
These fold envelopes (triangular grids) limit the maximum length of subsequences (black dots), while the alignment envelope (rectangular grid) limits the maximum deviation of cutpoints (short diagonal lines) from the main diagonal.

**Figure 5:**
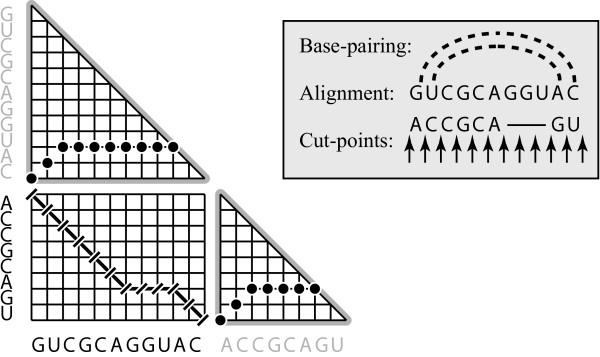
These fold envelopes (triangular grids) and alignment envelope (rectangular grid) limit the subsequences (black dots) and cutpoints (short diagonal lines) to those consistent with a given alignment and consensus secondary structure (shown). The alignment path is also shown on the alignment envelope as a solid black line, broken by cutpoints.

**Figure 6:**
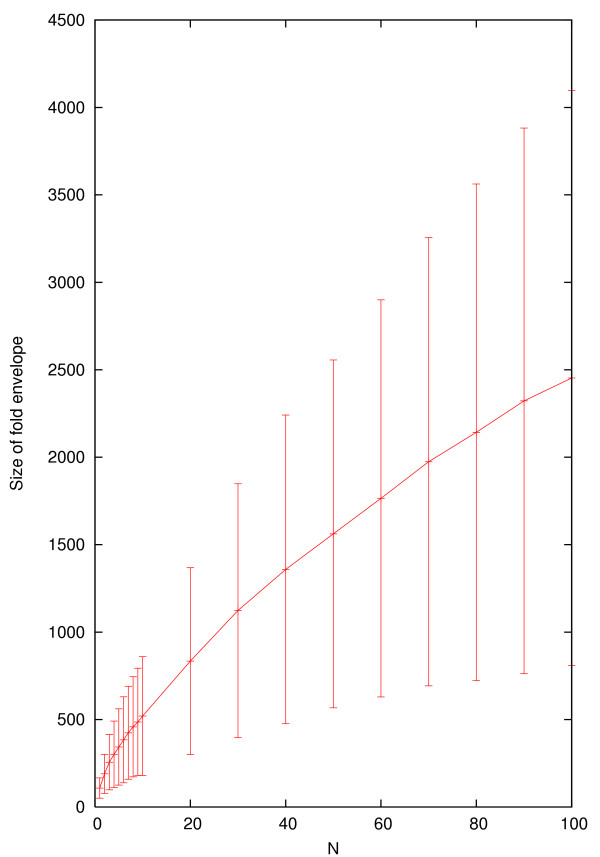
Fold envelope size is highly correlated with *N *in the *N*-best fold test, although the variance is large due to the diversity of alignments in the test.

**Figure 7:**
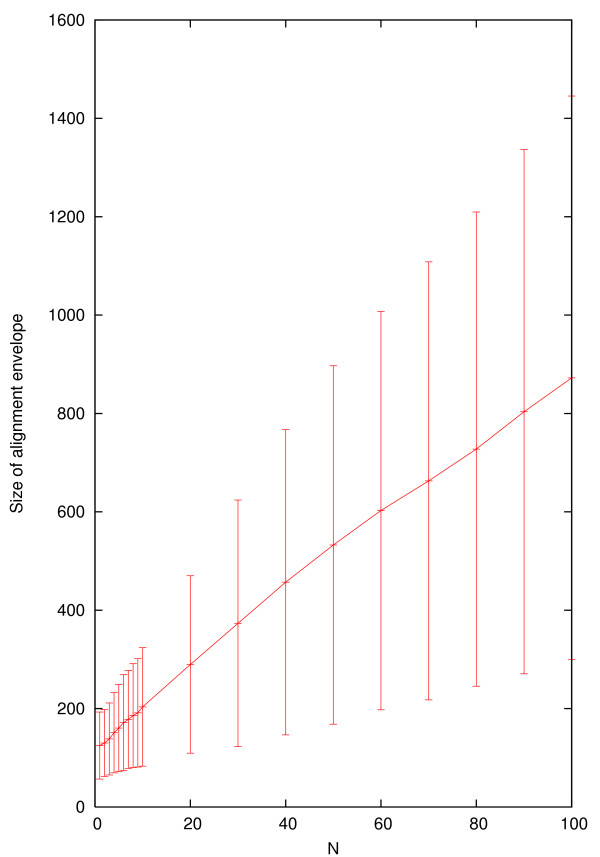
Alignment envelope size is highly correlated with *N *in the *N*-best alignment test, although the variance is large due to the diversity of alignments in the test.

**Figure 8:**
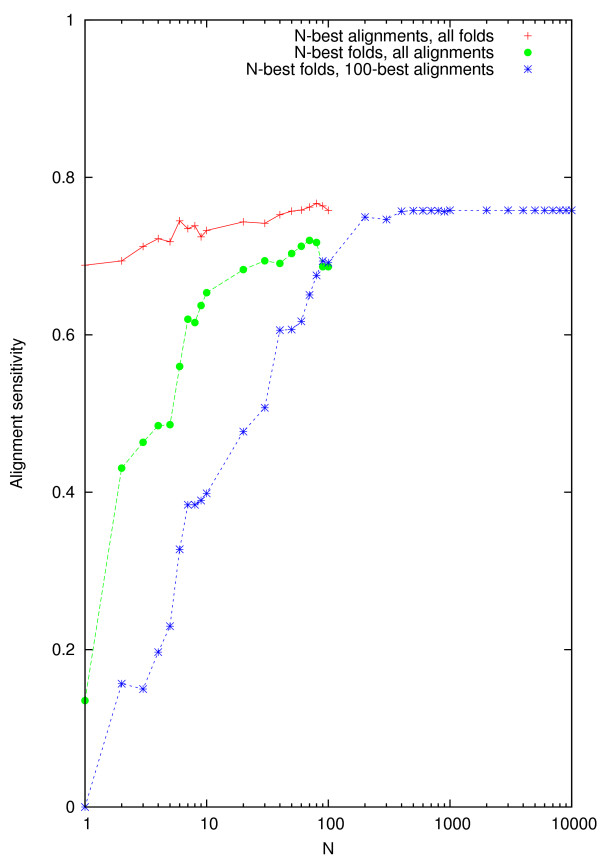
Alignment sensitivity as a function of envelope size parameter *N *for three different test regimes.

**Figure 9:**
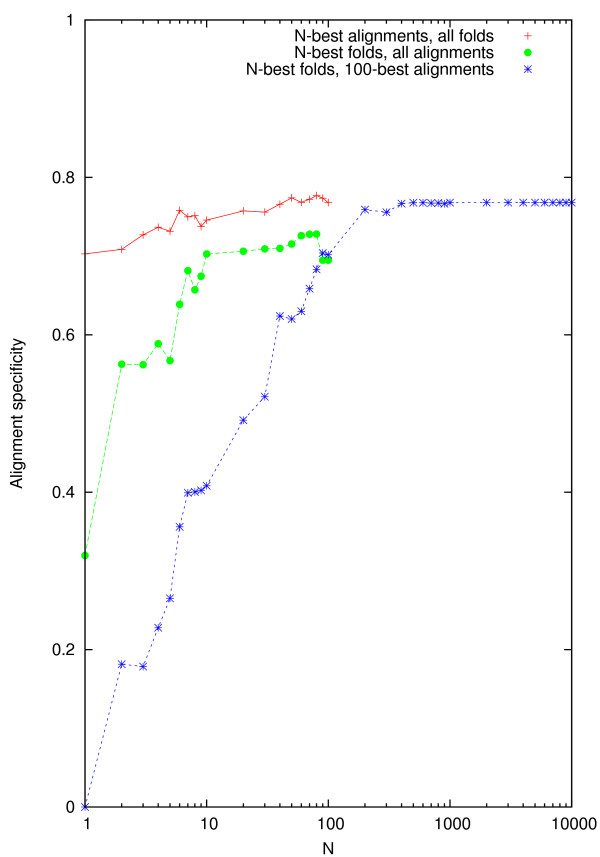
Alignment specificity as a function of envelope size parameter *N *for three different test regimes.

**Figure 10:**
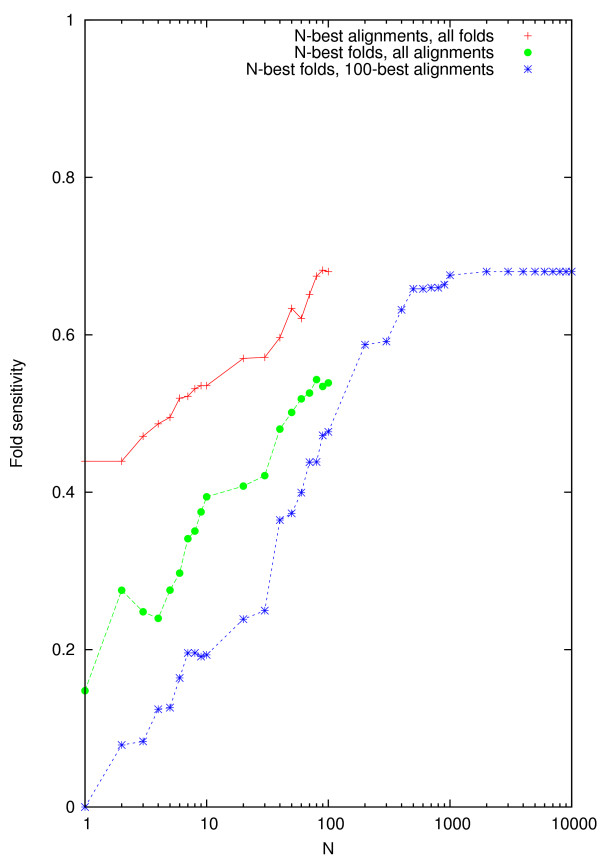
Fold sensitivity as a function of envelope size parameter *N *for three different test regimes.

**Figure 11:**
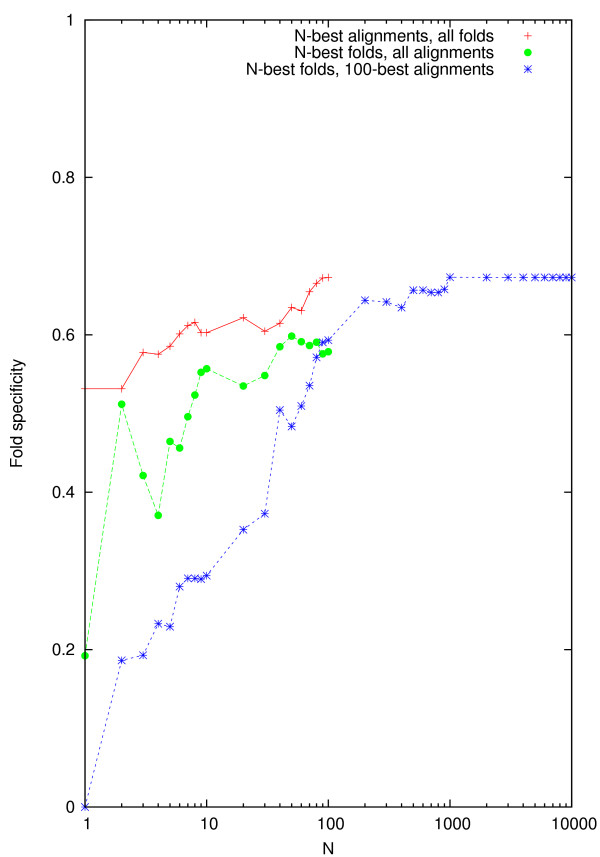
Fold specificity as a function of envelope size parameter *N *for three different test regimes.

**Figure 12:**
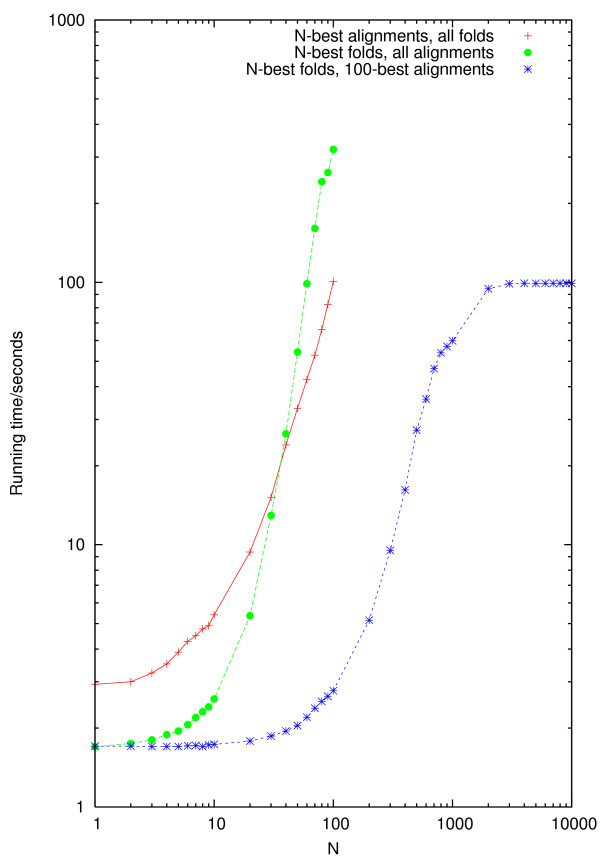
Total running time of stemloc (including envelope generation phases) as a function of envelope size parameter *N *for three different test regimes.

**Figure 13:**
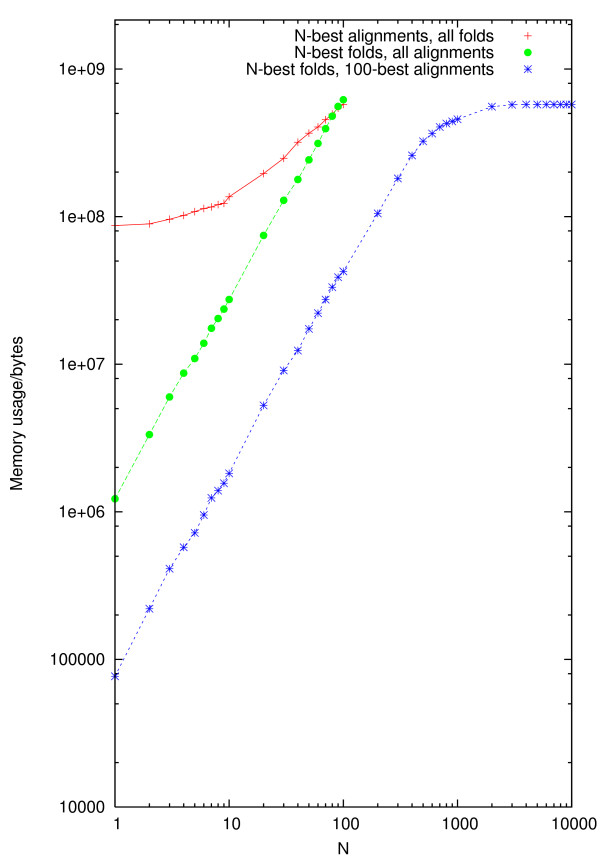
Peak memory usage of stemloc (i.e. the size of the principal CYK matrix) as a function of envelope size parameter *N *for three different test regimes.

As an alternative to the unconstrained Inside algorithm, we can partially initialize the envelopes to limit the maximum subsequence length and/or the maximum deviation of the alignment from the main diagonal (Figure 4). More flexibly, we can limit the recursion to a single alignment, a single structure, or a broadly-specified set of alignments or structures (Figure 5). Applications such as alignment of two known structures [13,32], alignment of an unstructured sequence to a known structure [33] or structure prediction from a known alignment [9] all reduce to simple application of the appropriate constraints.

#### Further possible constraints

The constraints given here allow the independent imposition of alignment or fold constraints. One can imagine further, even more general constraints. For example, one could exclude subsequence-pairs (*X*_*ij*_, *Y*_*kl*_) of radically different lengths, i.e. for which |(*j *- *i*) - (*k *- *l*)| exceeds some cutoff. This constraint is employed by the FOLDALIGN program. It is not expressible as a combination of independent alignment and fold constraints, and has not been implemented for the present work, though it would be relatively straightforward to combine it with the other constraints described here [10].

#### Accelerating the iteration

Simply setting some intermediate probabilities to zero is not sufficient to accelerate the Inside algorithm. We also need to redesign the iteration to avoid visiting zero-probability subsequence-pairs (*X*_*ij*_, *Y*_*kl*_). This is achieved by pre-indexing the fold envelopes ,  and the alignment envelope  so that we can quickly locate valid co-ordinates (*i*, *j*, *k*, *l*). The following is pseudocode for the algorithm with the redesigned iterator

• Inputs: *X*, *Y*, *S*, 

• For *i *= |*X*| to 0 (descending)

    • For each *j *satisfying (*i*, *j*) ∈  (ascending) (†)

       • For each *k *satisfying (*i*, *k*) ∈  (descending) (†)

          • For each *l *satisfying (*k*, *l*) ∈  (ascending) (†)

             • If (*j*, *l*) ∈  then

                • For each nonterminal *U *∈ Φ

                   • Set *Q*_1 _← 0; calculate *Q*_2_

                   • For each *m *satisfying {(*i*, *m*), (*m*, *j*)} ⊂  (†)

                      • For each *n *satisfying {(*k*, *n*), (*n*, *l*)} ⊂  (†)

                         • If (*m*, *n*) ∈  then

                            • Calculate *Q*_*B*_(*m*, *n*) and add to *Q*_1_

                   • Calculate (*i*, *j*, *k*, *l*) and store

• Return (0, |*X*|, 0, |*Y*|)

(†) These ordered subsets of ,  and  can be precomputed for speed.

Alternative designs for the algorithm are possible, and indeed different circumstances may affect the choice of optimal design (e.g. depending on which envelopes are most constrained).

#### Slimming the container

Memory is the most prohibitively expensive resource demand of the Inside algorithm. In its simplest form, the algorithm stores the intermediate probabilities (*i*, *j*, *k*, *l*) using a five-dimensional array indexed by *U*, *i*, *j*, *k *and *l*. To get the most benefit out of imposing constraints, it is necessary to replace this multidimensional array with an efficiently-indexed reduced-space container.

This design decision involves a close trade-off between CPU and memory usage. Initially, we tested various combinations of generic containers with *O*(*N*)-storage and *O*(*N *log *N*) access-times, such as balanced search trees [22]. These were found to be unbearably slow, and so we settled on a configuration of nested preindexed arrays. This configuration wastes some space, but has the important advantage of constant access time for given indices (*U*, *i*, *j*, *k*, *l*).

For fold envelope , we precompute and sort the list  = {(*i*, *j*)} ⊆  of subsequences starting at each position *i*. For each subsequence (*i*, *j*) ∈ , let  be its rank in . These  are also precomputed and stored. Similar precomputed sets  and ranks  are stored for .

Our DP matrix then uses an inner two-dimensional array nested inside an outer two-dimensional array.

The outer array has dimensions (|*X*|, |*Y*|) and is indexed by (*i*, *k*). The inner array has dimensions (||, ||) and is indexed by (, ). Cells of this inner array are further sub-indexed by nonterminal *U *using a standard fixed-length array, yielding (*i*, *j*, *k*, *l*).

This particular configuration is efficient when the alignment envelope is densely populated and the fold envelopes are sparsely populated. As with the redesigned iterator, there may be alternative designs that are resource-optimal under various different circumstances, depending on the nature of the envelopes.

#### The CYK algorithm

The Cocke-Younger-Kasami (CYK) dynamic programming algorithm [20] is related to the Inside algorithm, but replaces "*p *+ *q*" with "max(*p*, *q*)" in all formulae (that is, instead of summing probabilities, the maximum probability is always taken). The entries of the DP matrix, (*i*, *j*, *k*, *l*), represent the maximum likelihood of any parse tree for (*X*_*ij*_, *Y*_*kl*_). A recursive/stack-based traceback algorithm can be used to recover this parse tree, beginning from subsequence (*i*, *j*, *k*, *l*) = (0, |*X*|, 0, |*Y*|) (for global alignment) or (*i*, *j*, *k*, *l*) = argmax (*i*, *j*, *k*, *l*) (for local alignment) [13].

#### The Outside and KYC algorithms

The Outside and KYC algorithms widen the applications of probabilistic inference with SCFGs. The Outside algorithm, together with the Inside, can be used to recover posterior probabilities of given basepairs/columns, which can be used as alignment reliability indicators or as update counts in Expectation Maximization parameter training [20,26]. The KYC algorithm can be used with CYK to recover maximum-likelihood tracebacks from given co-ordinates. (We introduce the name "KYC" as a simple reversal of "CYK", reflecting the fact that KYC is to CYK as Outside is to Inside, i.e. the "reverse" version of the algorithm.)

These algorithms use dynamic programming recursions that are related to Inside and CYK. The Outside algorithm calculates intermediate probabilities of the form

(*i*, *j*, *k*, *l*) = *P*(*X*_0,*i*_, *Y*_0,*k*_, *V*, *Y*_*l*,|*Y*|_, *X*_*j*,|*X*| _|*S*)

representing the sum-over-probabilities of all partial parse trees rooted at *S *and ending in *V *without having yet generated sequences *X*_*ij *_and *Y*_*kl*_. Then, for example, the posterior probability that some node *V *in the parse tree has inside sequence-pair (*X*_*ij*_, *Y*_*kl*_) is



As CYK is related to Inside, the KYC algorithm is related to the Outside algorithm: the intermediate probabilities (*i*, *j*, *k*, *l*) are found using similar recursions (see below), but with all sums "*p *+ *q*" replaced by maxima "max(*p*, *q*)". The CYK and KYC DP matrices can be used to find the maximum likelihood of any parse tree containing a node *V *with inside sequence-pair (*X*_*ij*_, *Y*_*kl*_): this maximum likelihood is (*i*, *j*, *k*, *l*) (*i*, *j*, *k*, *l*). A recursive/stack-based traceback algorithm can be used to find the parse tree with this maximum likelihood.

As with the Inside algorithm, we sum contributions to (*i*, *j*, *k*, *l*) from various matching production rules. In contrast to the Inside algorithm, the nonterminal *V *that indexes  (...) must now be matched on the right-hand-side, not the left-hand-side, of these production rules. We first consider bifurcations from a source nonterminal *U *that adjoin adjacent subsequences to left (*U *→ *WV*) or right (*U *→ *VW*), so that the inside sequence-pairs for *U *are (respectively) (*X*_*mj*_, *Y*_*nl*_) or (X_*im*_,*Y*_*kn*_)



Next we consider emission rules, , again representing transitions as the special case *A' *= *B' *= *C' *= *D' *= 



The intermediate Outside probabilities are thus

(*i*, *j*, *k*, *l*) =  + 

(0, |*X*|, 0, |*Y*|) = 1 (Termination condition)

Note that the Inside probabilities (*i*, *j*, *k*, *l*) are needed to compute the Outside probabilities. We supply the Inside matrix, *I*, as an input to the Outside algorithm (they are usually calculated at the same time anyway).

In terms of the underlying iteration, the key difference between the Inside and Outside algorithms is as follows. Suppose subsequence-pair INNER = (*X*_*ij*_, *Y*_*kl*_) is enclosed by subsequence-pair OUTER = (*X*_*i'j'*_, *Y*_*k'l'*_) (that is, 0 ≤ *i' *≤ *i *≤ *j *≤ *j' *≤ |*X*| and 0 ≤ *k' *≤ *k *≤ *l *≤ *l' *≤ |*Y*|, and INNER ≠ OUTER). Then the Inside iterator visits INNER before OUTER, whereas the Outside iterator visits OUTER before INNER. The order in which the Outside iterator visits nonterminals is topologically forward-sorted with respect to the grammar's transition-rule graph (i.e. the reverse of the order used by the Inside iterator).

• Inputs: *X*, *Y*, *S*, , *I*

• Initialize (0, |*X*|, 0, |*Y*|)

• For *i *= 0 to |*X*| (ascending)

    • For each *j *satisfying (*i*, *j*) ∈  (descending) (†)

       • For each *k *satisfying (*i*, *k*) ∈  (ascending) (†)

          • For each *l *satisfying (*k*, *l*) ∈  (descending) (†)

             • If (*j*, *l*) ∈  then

                • For each nonterminal *U *∈ Φ (reverse order)

                   • Set  ← 0; calculate 

                   • For each *m *satisfying {(*m*, *j*), (*m*, *i*)} ⊂  (†)

                      • For each *n *satisfying {(*n*, *l*), (*n*, *k*)} ⊂  (†)

                         • If (*m*, *n*) ∈  then

                            • Calculate (*m*, *n*) and add to 

                   • For each *m *satisfying {(*i*, *m*), (*j*, *m*)} ⊂  (†)

                      • For each *n *satisfying {(*k*, *n*), (*l*, *n*)} ⊂  (†)

                         • If (*m*, *n*) ∈  then

                            • Calculate (*m*, *n*) and add to 

                   • Calculate (*i*, *j*, *k*, *l*) and store

(†) These ordered subsets of ,  and  can be precomputed for speed.

The reduced-space dynamic programming matrix that was developed above for the constrained Inside algorithm can be re-used for the constrained Outside algorithm.

### Implementation

The above-described algorithms were implemented in the C++ dart library. One dart program in particular, stemloc, is an efficient general-purpose RNA multiple-sequence alignment program that can be flexibly controlled by the user from the Unix command line, including re-estimation of parameters from training data as well as a broad range of alignment functions.

The dart libraries provide Inside, Outside, CYK, KYC, traceback and training algorithms for any pairwise SCFG in RNA normal form, whose rule probabilities can be expressed as algebraic expressions of some set of probability parameters (with associated normalization constraints). The operator-overloading features of C++ are utilized in full, so that the syntax of initializing a grammar object involves very few function calls and is essentially declarative.

dart source code releases can be downloaded under the terms of the GNU Public License, from the following URL (which also gives access to the latest development code in the CVS repository)



The grammars and algorithms described in this paper specifically refer to release 0.2 of the dart package, dated October 2004 (although the algorithms are also implemented in release 0.1, dated 10/2003).

#### Selecting appropriate fold and alignment envelopes

This section offers a non-exhaustive list of possible strategies for choosing appropriate *fold/***alignment **envelopes. *(Italicized terms *apply to fold envelopes, and **bold terms **to alignment envelopes.)

• Choose some appropriately simplified grammar, such as a *single-sequence SCFG/***pair HMM **that models *RNA folding***/primary sequence alignment. **Compute posterior probabilities of *subsequences*/**cutpoints **using the *Inside-Outside*/**Forward-Backward **algorithm for some *single-sequence SCFG/***pair HMM **that models *RNA folding*/**primary sequence alignment. **Select all *subsequences*/**cutpoints **with posterior probability above some threshold (or select e.g. the top 10 percentile of the posterior probabability distribution). Ensure that each of these *subsequences*/**cutpoints **is on a valid traceback path, e.g. by running the *CYK-KYC*/**Viterbi-forward-backward **algorithm to find the maximum-likelihood traceback path from any given *subsequence/***cutpoint**. Take the union of all *subsequences*/**cutpoints **on these traceback paths to obtain the required envelope.

• As above, choose some appropriate *single-sequence SCFG*/**pair HMM **that models *RNA folding */**primary sequence alignment**. Compute the maximum traceback path-likelihood from all *subsequences*/**cutpoints **using the *CYK-KYC*/**Viterbi-forward-backward **algorithm for this grammar. Select all *subsequences*/**cutpoints **with maximum traceback likelihood above some threshold (or select e.g. the top 10 percentile of the max-traceback likelihood distribution) and do a traceback from each such cell. Take the union of all *subsequences*/**cutpoints **on these traceback paths to obtain the required envelope.

• As above, choose some appropriate *single-sequence SCFG*/**pair HMM **that models *RNA folding */**primary sequence alignment**. Sample some number of *RNA structures ***/pairwise alignments **using the *Inside*/**Forward **algorithm with stochastic traceback. Take the union of all *subsequences*/**cutpoints **on these traceback paths to obtain the required envelope.

The latter two strategies have been implemented in the stemloc package described below. Empirically, the stochastic strategy appears to be less reliable than the deterministic strategies (although in theory the stochastic strategy will eventually find the globally optimal alignment given sufficiently many random repetitions, which may be a useful property).

#### Multiple sequence alignment

A heuristic algorithm for performing multiple alignment-and-folding of RNA sequences with a pairwise SCFG by progressive single-linkage clustering runs as follows

• Start by making pairwise alignments (with predicted secondary structures) for all pairs of input sequences.

• Mark the highest-scoring pair, and extract the two marked sequences *with their predicted secondary structures*. This highest-scoring pair is called the *seed alignment*.

• While some sequences remain unmarked:

    • For each newly-marked sequence:

       • Align the marked sequence, *with a fold envelope constrained by its predicted structure*, to each unmarked sequence in turn. (The fold envelope can be tailored to allow e.g. extension of local alignments.)

    • Select the highest-scoring of the pairwise (marked-to-unmarked) alignments. Use this alignment to merge the unmarked sequence into the seed alignment, and mark this sequence as newly aligned.

• Return the seed alignment.

The above algorithms have been implemented in stemloc. The multiple alignments produced by this algorithm lack well-defined probabilistic scores unless the pair SCFG is conditionally normalized. It is also straightforward to retrieve the *N *best non-overlapping alignments by repeatedly applying an incremental Waterman-Eggert-style mask to the alignment envelope [24]. This is implemented in stemloc for the pairwise case.

#### A grammar for pairwise RNA alignment and structure prediction

After some empirical experimentation, we developed the grammar of Tables 2, 3, 4 for the stemloc program. The grammar is split over three tables due to its considerable number of rules. Table 2 contains rules describing the connectivity of stems, loops and multiloops, and contains the only bifurcation rule. Table 3 describes the connectivity of bulges and Table 4 handles emissions (basepaired, unpaired, aligned or gapped).

The starting nonterminal is Start. The nonterminals representing higher-level units of RNA structure are Loop, Stem, LBulge, RBulge and LRBulge. Each of these has associated Match, Ins and Del states (e.g. StemMatch, StemIns and StemDel) and each of these states has an associated emission state, prefixed with *x*, *y *or *xy *superscripts (e.g. ^*xy*^StemMatch, ^*y*^StemIns and ^*x*^StemDel). The Multi state models multiloops, using a bifurcation to LMulti and RMulti.

Tables 2, 3, 4 also refer to probabilistic parameters used by the models. Free probability parameters (allowed to range from 0 to 1) include startInStem (determining the probability of ending the alignment with a basepair), loopExtend, stemExtend and multiExtend (determining the geometric distribution over loop and stem lengths, or the number of branches in a multiloop), multiBulgeOpen (determining the probability of bulges in multiloops) and stemGapOpen, stemGapExtend, stemGapSwap, loopGapOpen, loopGapExtend and loopGapSwap (determining the geometric distribution over gap lengths in stems and loops). There are also five parameter arrays: postStem[4] (determining whether a stem is followed by a loop, a bulge, a double-stranded bulge or a multiloop); baseIndel[4] (a probability distribution for a single unaligned, unpaired nucleotide); baseSubstitution[16] (a joint probability distribution for two aligned, unpaired nucleotides); basepairIndel[16] (a joint probability distribution for two unaligned, basepaired nucleotides); and basepairSubstitution [256] (a joint probability distribution for four aligned, basepaired nucleotides). All of the above parameters were automatically estimated from training data by the dart software.

To summarize, the grammar models homologous stems, loops, multiloops and bulges in pairwise RNA alignments, with covariant substitution scores and affine gap penalties (geometric indel length distributions). It has the property that any combined alignment and structure prediction for two RNA sequences has a single, unambiguous parse tree. In our investigations, this unambiguity appeared to improve the accuracy of alignment and structure prediction substantially; see also writings on this topic by Giegerich [34] and Dowell, Eddy *et al *[35]. The grammar is also designed to minimize the number of bifurcation rules (the only bifurcation is Multi → LMulti RMulti).

The stemloc grammar does not model basepair stacking effects due to *π*-orbital overlap, nor does it allow low-cost insertion or deletion of whole stems or substructures. Since the algorithms are implemented for any SCFG, it is straightforward to modify the program to experiment with grammars that model such phenomena. An example of a grammar that models the latter type of mutation (whole-substructure indels), and is also fully derived from an evolutionary rate-based model, is presented in a companion paper [36].

#### Parameterization

Under the SCFG framework, the probability parameters for the grammar can be estimated directly from data using the Inside-Outside algorithm with appropriate constraints, which are easy to supply (e.g. to sum over all parses consistent with a given alignment, one simply uses an appropriate alignment envelope). The parameters were trained from 56376 (non-independent) pairwise alignments from RFAM [37], with each pairwise alignment making a weighted contribution of 1/*N *to the counts computed during Expectation Maximization training, where *N *is the number of sequences in the multiple alignment from which the pairwise alignments were derived. Furthermore, the pairwise alignments were binned according to sequence identity, providing four alternative parameterisations; the bin ranges were 30–40%, 50–60%, 70–80% and 90–100%.

stemloc allows the user to re-estimate all parameters from their own personal training set of trusted alignments. This may be a useful feature, since the training procedure described above is probably biased. Since training was performed using all kinds of sequence available in RFAM, including RNA sequences with computationally predicted secondary structure as well as those for which structures were experimentally confirmed, it is possible that the stemloc parameters may be skewed by the parameters of the computational methods used by the RFAM curators to predict structure. These include the homology modeling program INFERNAL [37] and the *de novo *structure prediction program PFOLD [8].

## Authors' contributions

IH designed, programmed, tested and documented the algorithms.

## Figures and Tables

**Table 1:** A stochastic context-free grammar for generating pairwise alignments of RNA structures.

*L*	→	**R**	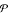 (*L *→ **R**)
Stem	→	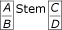	stemExtend (1 - stemGap) basepairSubstitution [*AC*, *BD*]
	|	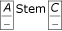	stemExtend (stemGap/2) basepairIndel [*AC*]
	|	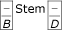	stemExtend (stemGap/2) basepairIndel [*BD*]
	|	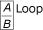	(1 - stemExtend)(1 - bifurcate) baseSubstitution [*AB*]
	|	Stem Stem	(1 - stemExtend) bifurcate
Loop	→	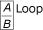	loopExtend (1 - loopGap) baseSubstitution [*AB*]
	|	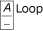	loopExtend (loopGap/2) baseIndel [*A*]
	|	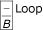	loopExtend (loopGap/2) baseIndel [*B*]
	|	*ε*	1 - loopExtend

**Table 2:** The stemloc grammar, part 1 of 3: stem and loop structures.

*L*	→	**R**	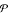 (*L *→ **R**)
Start	→	Stem	startInStem
	|	LBulge	(1 - startInStem) postStem [2]/ (2 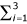 postStem[*i*])
	|	RBulge	(1 - startInStem) postStem[2]/ (2 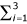 postStem[*i*])
	|	LRBulge	(1 - startInStem) postStem [3]/ ( 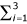 postStem[*i*])
	|	Multi	(1 - startInStem) postStem [4]/ ( 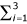 postStem[*i*])
Stem	→	^*xy*^StemMatch	1 - stemGapOpen
	|	^*y*^StemIns	stemGapOpen/2
	|	^*x*^StemDel	stemGapOpen/2
StemMatch	→	^*xy*^StemMatch	(1 - stemGapOpen) stemExtend
	|	^*y*^StemIns	stemGapOpen/2
	|	^*x*^StemDel	stemGapOpen/2
	|	StemExit	(1 - stemGapOpen)(1 - stemExtend)
StemIns	→	^*xy *^StemMatch	(1 - stemGapExtend)(1 - stemGapSwap) stemExtend
	|	^*y*^StemIns	stemGapExtend
	|	^*x*^StemDel	(1 - stemGapExtend) stemGapSwap
	|	StemExit	(1 - stemGapExtend) (1 - stemGapSwap)(1 - stemExtend)
StemDel	→	^*xy*^StemMatch	(1 - stemGapExtend)(1 - stemGapSwap) stemExtend
	|	^*x*^StemDel	stemGapExtend
	|	^*y*^StemIns	(1 - stemGapExtend) stemGapSwap
	|	StemExit	(1 - stemGapExtend) stemGapSwap (1 - stemExtend)
StemExit	→	Loop	postStem [1]
	|	LBulge	postStem [2]/2
	|	RBulge	postStem [2]/2
	|	LRBulge	postStem [3]
	|	Multi	postStem [4]
Multi	→	LMulti RMulti	1
LMulti	→	LBulge	multiBulgeOpen
	|	Stem	(1 - multiBulgeOpen)
RMulti	→	Multi	multiExtend
	|	Stem	(1 - multiExtend)(1 - multiBulgeOpen)^2^
	|	LBulge	(1 - multiExtend)(1 - multiBulgeOpen) multiBulgeOpen
	|	RBulge	(1 - multiExtend)(1 - multiBulgeOpen) multiBulgeOpen
	|	LRBulge	(1 - multiExtend) multiBulgeOpen^2^
Loop	→	^*xy*^LoopMatch	(1 - loopGapOpen)
	|	^*y*^LoopIns	loopGapOpen/2
	|	^*x*^LoopDel	loopGapOpen/2
LoopMatch	→	^*xy*^LoopMatch	(1 - loopGapOpen) loopExtend
	|	^*y*^LoopIns	loopGapOpen/2
	|	^*x*^LoopDel	loopGapOpen/2
	|	*ε*	(1 - loopGapOpen) (1 - loopExtend)
LoopIns	→	^*xy*^LoopMatch	(1 - loopGapExtend)(1 - loopGapSwap) loopExtend
	|	^*y*^LoopIns	loopGapExtend
	|	^*x*^LoopDel	(1 - loopGapExtend) loopGapSwap
	|	*ε*	(1 - loopGapExtend)(1 - loopGapSwap) (1 - loopExtend)
LoopDel	→	^*xy*^LoopMatch	(1 - loopGapExtend)(1 - loopGapSwap) loopExtend
	|	^*x*^LoopDel	loopGapExtend
	|	^*y*^LoopIns	(1 - loopGapExtend) loopGapSwap
	|	*ε*	(1 - loopGapExtend)(1 - loopGapSwap)(1 - loopExtend)

**Table 3:** The stemloc grammar, part 2 of 3: bulges.

*L*	→	**R**	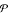 (*L *→ **R**)
LBulge	→	^*xy*^LBulgeMatch	(1 - loopGapOpen)
	|	^*y*^LBulgeIns	loopGapOpen/2
	|	^*x*^LBulgeDel	loopGapOpen/2
LBulgeMatch	→	^*xy*^LBulgeMatch	(1 - loopGapOpen) loopExtend
	|	^*y*^LBulgeIns	loopGapOpen/2
	|	^*x*^LBulgeDel	loopGapOpen/2
	|	Stem	(1 - loopGapOpen)(1 - loopExtend)
LBulgeIns	→	^*xy*^LBulgeMatch	(1 - loopGapExtend)(1 - loopGapSwap) loopExtend
	|	^*y*^LBulgeIns	loopGapExtend
	|	^*x*^LBulgeDel	(1 - loopGapExtend) loopGapSwap
	|	Stem	(1 - loopGapExtend)(1 - loopGapSwap) (1 - loopExtend)
LBulgeDel	→	^*xy*^LBulgeMatch	(1 - loopGapExtend)(1 - loopGapSwap) loopExtend
	|	^*x*^LBulgeDel	loopGapExtend
	|	^*y*^LBulgeIns	(1 - loopGapExtend) loopGapSwap
	|	Stem	(1 - loopGapExtend)(1 - loopGapSwap) (1 - loopExtend)
RBulge	→	^*xy*^RBulgeMatch	(1 - loopGapOpen)
	|	^*y*^RBulgeIns	loopGapOpen/2
	|	^*x*^RBulgeDel	loopGapOpen/2
RBulgeMatch	→	^*xy*^RBulgeMatch	(1 - loopGapOpen) loopExtend
	|	^*y*^RBulgeIns	loopGapOpen/2
	|	^*x*^RBulgeDel	loopGapOpen/2
	|	Stem	(1 - loopGapOpen) (1 - loopExtend)
RBulgeIns	→	^*xy*^RBulgeMatch	(1 - loopGapExtend)(1 - loopGapSwap) loopExtend
	|	^*y*^RBulgeIns	loopGapExtend
	|	^*x*^RBulgeDel	(1 - loopGapExtend) loopGapSwap
	|	Stem	(1 - loopGapExtend)(1 - loopGapSwap) (1 - loopExtend)
RBulgeDel	→	^*xy*^RBulgeMatch	(1 - loopGapExtend)(1 - loopGapSwap) loopExtend
	|	^*x*^RBulgeDel	loopGapExtend
	|	^*y*^RBulgeIns	(1 - loopGapExtend) loopGapSwap
	|	Stem	(1 - loopGapExtend)(1 - loopGapSwap) (1 - loopExtend)
LRBulge	→	^*xy*^LRBulgeMatch	(1 - loopGapOpen)
	|	^*y*^LRBulgeIns	loopGapOpen/2
	|	^*x*^LRBulgeDel	loopGapOpen/2
LRBulgeMatch	→	^*xy*^LRBulgeMatch	(1 - loopGapOpen) loopExtend
	|	^*y*^LRBulgeIns	loopGapOpen/2
	|	^*x*^LRBulgeDel	loopGapOpen/2
	|	RBulge	(1 - loopGapOpen) (1 - loopExtend)
LRBulgeIns	→	^*xy*^LRBulgeMatch	(1 - loopGapExtend)(1 - loopGapSwap) loopExtend
	|	^*y*^LRBulgeIns	loopGapExtend
	|	^*x*^LRBulgeDel	(1 - loopGapExtend) loopGapSwap
	|	RBulge	(1 - loopGapExtend)(1 - loopGapSwap) (1 - loopExtend)
LRBulgeDel	→	^*xy*^LRBulgeMatch	(1 - loopGapExtend)(1 - loopGapSwap) loopExtend
	|	^*x*^LRBulgeDel	loopGapExtend
	|	^*y*^LRBulgeIns	(1 - loopGapExtend) loopGapSwap
	|	RBulge	(1 - loopGapExtend)(1 - loopGapSwap) (1 - loopExtend)

**Table 4:** The Stemloc grammar, part 3 pf 3: emissions.

*L*	→	**R**	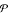 (*L *→ **R**)
^*xy*^LoopMatch	→	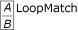	baseSubstitution [*A*, *B*]
^*y*^LoopIns	→	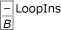	baseIndel [*B*]
^*x*^LoopDel	→	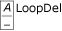	baseIndel [*A*]
^*xy*^LBulgeMatch	→	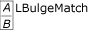	baseSubstitution [*A*, *B*]
^*y*^LBulgeIns	→	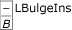	baseIndel [*B*]
^*x*^LBulgeDel	→	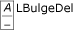	baseIndel [*A*]
^*xy*^RBulgeMatch	→	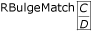	baseSubstitution [*C*, *D*]
^*y*^RbulgeIns	→	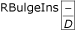	baseIndel [*D*]
^*x*^RBulgeDel	→	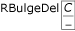	baseIndel [*C*]
^*xy*^LRBulgeMatch	→	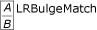	baseSubstitution [*A*, *B*]
^*y*^LRBulgeIns	→	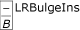	baseIndel [*B*]
^*x*^LRBulgeDel	→	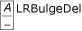	baseIndel [*A*]
^*xy*^StemMatch	→	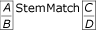	basepairSubstitution [*AC*, *BD*]
^*y*^StemIns	→	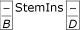	basepairIndel [*BD*]
^*x*^StemDel	→	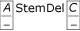	basepairIndel [*AC*]

**Table 5:** The subset of RFAM used to test the constrained SCFG algorithms.

RFAM family	Seauence (EMBL.ID / startpoint-endpoint)		Alignment		Basepair	
	*X*	*Y*	sens.	spec.	sens.	spec.
S15	AE004150.1/7123-7243	AE004888.1/2785-2659	0.65	0.752	0.462	0.353
S15	AE005545.1/3797-3683	AE004888.1/2785-2659	0.652	0.701	0.615	0.4
U3	U27297.1/2-180	AF277396.1/3-126	0.252	0.248	0.0833	0.087
glmS	AL935254.1/94449-94600	AE010557.1/24-169	0.603	0.599	0.667	0.595
glmS	AE010557.1/24-169	AE013165.1/2616-2459	0.532	0.587	0.545	0.667
glmS	AL596166.1/50734-50929	AE013165.1/2616-2459	0.873	0.873	0.757	0.7
glmS	AC078934.3/32621-32405	AE010557.1/24-169	0.869	0.863	0.756	0.689
glmS	AL935254.1/94449-94600	AE013165.1/2616-2459	0.715	0.715	0.811	0.769
Purine	AE007775.1/3558-3459	AL591981.1/205922-205823	0.869	0.869	0.773	0.81
Purine	AL591981.1/205922-205823	AP004595.1/160373-160472	0.838	0.838	0.591	0.5
Purine	AE007775.1/3558-3459	AE010606.1/4680-4581	0.67	0.699	0.636	0.875
Purine	AP003194.2/163700-163601	AE016809.1/202496-202595	0.84	0.866	0.857	0.75
U5	M16510.1/245-451	AF095839.1/890-777	0.584	0.579	0.667	0.8
U5	X63789.1/2236-2349	AF095839.1/890-777	0.716	0.69	0.8	0.8
IRE	AY112742.1/12-41	S57280.1/391-417	0.667	0.667	0.6	0.75
IRE	AF266195.1/14-43	X01060.1/3950-3976	0.963	0.963	0.9	0.9
IRE	S57280.1/391-417	X13753.1/1434-1460	1	1	0.6	0.6
IRE	AY112742.1/12-41	X13753.1/1434-1460	0.778	0.778	0.8	0.727
IRE	AF266195.1/14-43	AF171078.1/1416-1442	0.963	0.963	0.7	0.7
IRE	AF171078.1/1416-1442	X01060.1/3950-3976	1	1	0.7	0.875
6S	Y00334.1/77-254	AL627277.1/108623-108805	0.869	0.869	0.811	0.754
6S	AE004317.1/5626-5807	AL627277.1/108623-108805	0.777	0.777	0.736	0.709
